# Characterization of Crohn's disease patients in Egypt: Risk factors for postoperative recurrence (A cohort study)

**DOI:** 10.1016/j.amsu.2021.102781

**Published:** 2021-09-06

**Authors:** Shimaa Kamel, Mohamed Sakr, Waleed Hamed, Mohamed Eltabbakh, Ahmed Sherief, Heba Rashad, Yasser Elghamrini, Ahmed Elbaz

**Affiliations:** a Department of Tropical Medicine, Gastroenterology, and Hepatology, Ain Shams University, Abbasiya, Cairo, Egypt; b Department of General Surgery, Ain Shams University, Abbasiya, Cairo, Egypt

**Keywords:** Biological therapy, Characterizations, Crohn's disease, Montreal classification, Postoperative recurrence

## Abstract

**Background:**

The aim of study to identify the characterizations of Crohn's disease in Egyptian patients and to determine its predictors for postoperative recurrence.

**Methods:**

We conducted a retrospective observational cohort study on 15 patients diagnosed as Crohn's disease with surgical interventions. Different characteristics of studied patients were analyzed to determine the risk factors for postoperative recurrence such as age at diagnosis, gender, smoking, main presenting symptom, Montreal classification, perianal disease, laboratory findings and protocol of management including surgical characteristics like age at operation, surgical indication, preoperative medication, surgical approach, and operative findings.

**Results:**

Nine of the studied patients (60%) suffered from clinical postoperative recurrence with mean duration of 23.5 ± 40.6 months. In comparison the demographic, clinical, operative, and medical treatment data between patients with postoperative recurrence of Crohn's disease and those without recurrence, age at diagnosis (mean age 42.9 years) and age at operation (mean 44.7 years) were found statistically significant in postoperative recurrence group (p-value = 0.001). According to Montreal classification of Crohn's disease, patients >40 years were significantly found in postoperative recurrence group, while patients between 17 and 40 years were significantly found in postoperative non-recurrence group (p-value=0.007) and ileal location of Crohn's disease was found significantly in postoperative recurrent group (p-value=0.044). Postoperative biological therapy significantly decreased the incidence of postoperative recurrence in the current study (p-value= 0.041).

**Conclusions:**

Age at diagnosis, age at operation, ileal location of Crohn's disease can significantly predict postoperative recurrence. Also, postoperative biological therapy can significantly decrease the incidence of postoperative recurrence.

## Highlights

• Crohn's disease (CD) is a chronic inflammatory bowel disease with unclear etiology.
• Surgery is essential for its symptomatic control and treatment of complications.
• Postoperative recurrence is challenging in management of CD.
• In this study we determine the predictors for postoperative recurrence.

## 1 Introduction

Crohn's disease (CD) is a chronic inflammatory bowel disease (IBD), its etiology is unclear until now, but it was found to be correlated to different environmental conditions that could activate the disease in genetically susceptible people [1].

Many epidemiological studies showed high prevalence of CD in western developed countries, while few studies were reported from developing countries. This could be attributed to either difference of environmental conditions between these countries or absence of accurate registry for IBD patients [2,3].

In the last two decades, CD evolved and reported in different developing Middle East and North African countries associated with dramatical change of their lifestyle and diet habits by spreading of fast food and decrease intake of dietary fibers, increase of psychological stress, increase environmental pollution and decrease of parasitic infections. In Egypt, some tertiary centers have been evolved for management of IBD with precise patient registry [4].

CD is characterized by remitting and relapsing nature. Its lifelong treatment includes corticosteroids and immunomodulators [5,6]. In the last two decades the nature of the disease have been dramatically changed with the advent of biological therapy [7,8]. Surgery is essential in management of CD for symptomatic control and treatment of complications. Surgery is needed in 25%–30% of these patients within 5–10 years, respectively [5,6]. But postoperative recurrence is challenging in management of CD, as 25–45% of these patients will need another surgical operation within 10 years after the first surgical intervention [8,9].

The aim of current study was to identify the characterizations of CD in Egyptian patients and to determine its predictors for postoperative recurrence.

## 2 Patients and methods

### 2.1 Study design

We conducted a retrospective observational cohort study on patients who were referred to our IBD unit of Tropical Medicine Department Ain Shams University Hospitals, Cairo, Egypt, which is one of the largest tertiary hospitals serving patients from all areas in Egypt. Our work has been reported in line with the STROCSS criteria [10].

Our center is receiving about 700 patients per year for gastrointestinal consultations and/or interventions. Annually, approximately 60 patients are diagnosed as ulcerative colitis (UC) and 10 patients are diagnosed as CD. Our center offers all types of services including all the diagnosis modalities, all lines of treatment including the biological treatment. Our decisions were taken in multidisciplinary team meeting with colorectal surgeons, pathologists, and radiological consultants.

### 2.2 Study approval

The study was approved by Research Ethics Committee of Ain Shams University Faculty of Medicine corresponding to declaration of Helsinki principles (FMASU R 78/2021). All studied patients approved their involvement in the study by written, informed consent. The study was registered at research registry with unique identifying number (UIN) researchregistry7022.

### 2.3 Study population

Fifteen patients diagnosed as CD with surgical interventions were included in the current study collected from IBD database of 33 patients diagnosed as CD between 2015 and 2020.

Patients were excluded if they did not follow-up in our center after surgery, non-compliant to postoperative medications or data was missing.

### 2.4 Protocol of management

In our regular weekly clinics, our patients were diagnosed by clinical assessment, laboratory investigations e.g., erythrocyte sedimentation rate (ESR) and C-reactive protein (CRP), imaging procedures such as bowel ultrasonography and magnetic resonance enterography and confirmed with histopathologic examination of biopsies were taken during colonoscopy. These patients were followed up regularly by clinical assessment, laboratory investigations, bowel ultrasonography and magnetic resonance enterography every 3–6 months and colonoscopy every 1–5 years according to the degree of risk factors of the disease [11]. Full assessment of any case to be done at any time with acute exacerbation of the disease.

In our center, we use step-up approach in the management of our patients, we have used probiotics and antibiotics in the form of ciprofloxacin, metronidazole in cases with infection, and courses of steroid or budesonide in exacerbation, azathioprine in steroid dependent cases or resistant cases. Methotrexate was used in some selected cases. Also, we used biological therapy anti-tumor necrosis factor (TNF) after exclusion of infection or tuberculosis in resistant cases to ordinary treatment or in complicated fistulizing cases. Patients who were indicated for surgery received 4 weeks postoperatively medications according to our protocol of management; azathioprine 2.5 mg/kg in low-risk group and biological therapy in high-risk group [12].

### 2.5 Postoperative recurrence

Postoperative recurrence was either clinical and/or radiological. Clinical recurrence was defined as recurrence of symptoms e.g., abdominal pain, diarrhea, or fever due to disease activity (after exclusion of other causes) which could be confirmed by laboratory markers of activity or endoscopic findings suggesting recurrence (even at the anastomotic site) or development of fistula [12].

Radiological recurrence was defined as detection of signs of disease activity by bowel ultrasonography or MRE during follow-up of the patients such as thickening of bowel loops, increase doppler vascularity in suspected segment and enteroenteric or enterocutaneous fistula [11].

Different characteristics of studied patients were recorded and analyzed to determine the risk factors for postoperative recurrence such as age at diagnosis, gender, smoking, main presenting symptom, Montreal classification, perianal disease, laboratory findings and protocol of management including surgical characteristics like age at operation, surgical indication, preoperative medication, surgical techniques, and operative findings.

### 2.6 Statistical analysis

Data was analyzed by Statistical Package for Social Science (IBM SPSS) version 20. Mean and standard deviation represented quantitative data, while number and percentages represented qualitative data. Student's t-test was used to compare between quantitative data, while Fisher's exact test was used to compare between qualitative data. The p-value was considered significant if p-value < 0.05.

## 3 Results

Regarding the demographic data of studied patients of CD with surgical intervention, the mean age at diagnosis for the studied cases was 37.93 ± 7.86 years, and mean age at operation was 40.13 ± 7.65 years. Nine of them were female patients and non-smoker (60%) and 6 were male patients and smoker (40%). The main presenting symptoms were abdominal pain in 9 patients (60%), 6 patients presented with diarrhea (40%) and one patient with bleeding per rectum (6.7%). Studies patients were categorized according to Crohn's Disease Activity Index (CDAI) score as mild in 5 patients (33.3%) and moderate to severe in 10 patients (66.7%) and no patients were severely active or fulminant (0%). Concerning laboratory findings, mean of ESR was 58.6 ± 30.18 mm/h and mean of CRP was 38.31 ± 41.69 mg/L.

Studied patients were categorized according to Montreal Classification of CD defined three age categories: A1 if age <16 years, A2 if age 17–40 years, or A3 if age >40 years; four locations of CD: L1 in ileum, L2 in colon, L3 in ileocolon, or L4 in upper gastrointestinal tract; and three behaviors for CD: B1 if non-stricturing non-penetrating, B2 if stricturing, or B3 if penetrating. Penetrating behavior of CD was determined if patient developed in the course of his disease any intra-abdominal fistula, perforation of bowel, inflammatory mass or abscess [13]. Perianal and rectovaginal fistulas were not considered as penetrating disease. Patients were classified as B3 if they have both stricturing and penetrating behaviors according to Oberhuber et al. [14]. Montreal Classification of CD showed that 8 patients (53.3%) were ≤40 years, while 7 patients (46.7%) were >40 years. The location of lesions was ileal in 10 cases (66.7%), ileocolonic in 3 patients (20%) and colonic in 2 patients (13.3%). Behavior of CD was stricturing in 3 patients (20%) and penetrating in 12 patients (80%) while perianal disease was found only in 2 patients (13.3%). The endoscopic findings of studied patients showed that aphthous ulcers were found in 9 patients (60%), linear ulcers in 6 patients (40%), while 7 patients (46.6%) had cobble stone appearance.

Eleven patients (73.7%) received courses of steroid, 8 patients (53.5%) received azathioprine and 8 of studied patients (53.5%) received biological treatment. Regarding postoperative recurrence, 9 of studied patients (60%) suffered from clinical recurrence and 4 of them showed radiological recurrence also in the form of bowel thickening (≥5 mm) in one of the bowel segments as diagnosed by follow-up bowel ultrasonography. In current study the mean time of postoperative recurrence was 23.5 ± 40.6 months.

**Fig. 1:**
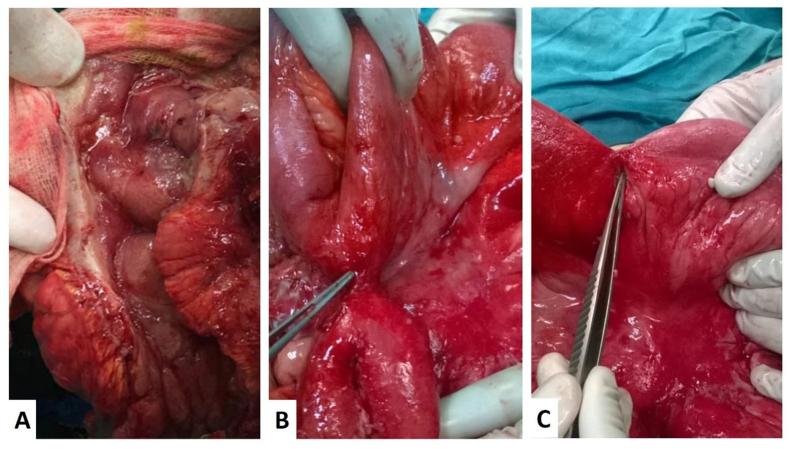
**(A)** Linear ulcers in between cobblestone of caecum of Crohn's disease patient. **(B)** Intraoperative view of enteroenteric fistula pointed by forceps. **(C)** Intraoperative picture demonstrating stretched-out small intestinal stricture with bowel obstruction indicated by surgical instrument.

Intraoperative findings of studied patients showed 9 patients had ileocecal stricture and 4 patients had small intestinal stricture. Six patients (40%) presented with intestinal obstruction. Small intestinal perforation was found in 3 patients (20%) all of them were in postoperative non-recurrent group. Inflammatory ileocecal mass was found in 6 patients (40%) (shown in Fig. 1). Right hemicolectomy was done for 9 patients (60%), sigmoid colectomy for 2 patients (13.3%), strictureplasty for one patient (6.6%). Three patients (20%) had small intestinal resection anastomosis with fistulectomy. Three patients (20%) have surgical history of appendectomy, and one patient (6.6%) had history of diagnostic laparoscopy. Two patients (13.3%) had previous surgery for CD, one of them had right hemicolectomy then 3 years later small intestinal resection for obstructing stricture, the other patient underwent stricturoplasty followed by right hemicolectomy 18 months later for obstructing ileocecal mass.

Postoperatively, 5 patients received azathioprine (33.4%) and 7 patients (46.6%) received anti-TNFs in the form of infliximab or adalimumab. Three patients (20%) were non-complaint on postoperative treatment.

Comparing the demographic, clinical, operative, and medical treatment data between patients with postoperative recurrence of CD (Group 1; G1) and those without recurrence (Group 2; G2), age at diagnosis with mean age 42.9 years and age at operation with mean 44.7 years were found significantly in G1 (p-value = 0.001). According to Montreal classification of CD, patients >40 years were significantly found in G1, while patients between 17 and 40 years were significantly found in G2 (p-value = 0.007). Also, current study showed that laboratory investigations and preoperative treatment had no statistically significant difference between both groups. Regarding the operative details in studied patients, ileal location of CD was statistically significant in G1 (p-value = 0.044). Otherwise, none of the type nor features of surgery could predict postoperative recurrence. Current results showed that postoperative biological therapy could significantly decrease the incidence of postoperative recurrence (p-value = 0.041). (Table 1).

**Table 1:** Comparison between different patients' parameters among studied groups.

Parameter	G1 (n = 9)	G2 (n = 6)	p-value
Gender [male/female]	4/5	2/4	1
Smoking [n (%)]	4 (44.4%)	2 (33.3%)	1
Age at diagnosis [mean ± SD]	42.9 ± 5.6	30.5 ± 3.2	0.001
Age at operation [mean ± SD]	44.7 ± 5.9	33.3 ± 3.9	0.001
Laboratory findings [mean ± SD]			
Hemoglobin [11.5–15.5 g/dL]	10.3 ± 1.5	10.0 ± 2.4	0.777
Platelets [150–450 10∧3/μL]	425.7 ± 190.1	308.5 ± 52.1	0.111
AST [up to 37 IU/L]	24.3 ± 11.0	24.7 ± 9.5	0.953
ALT [up to 40 IU/L]	22 ± 8.2	23.7 ± 6.7	0.687
Total proteins [6–8.3 g/dL]	6.7 ± 0.8	7.0 ± 0.7	0.438
Albumin [3.5–5 g/dL]	3.8 ± 0.6	3.6 ± 0.8	0.633
Total bilirubin [up to 1.2 mg/dL]	0.6 ± 0.3	0.8 ± 0.2	0.270
Direct bilirubin [up to 0.3 mg/dL]	0.3 ± 0.2	0.4 ± 0.2	0.212
INR	1.1 ± 0.1	1.1 ± 0.1	0.789
BUN [7–21 mg/dL]	9.2 ± 2.2	8.7 ± 1.0	0.527
Creatinine [0.4–1.3 mg/dL]	0.8 ± 0.2	0.7 ± 0.3	0.473
Sodium [136–146 mEq/L]	136.3 ± 2.1	136.2 ± 2.6	0.891
Potassium [3.5–5.2 mEq/L]	3.9 ± 0.3	3.7 ± 0.4	0.272
CRP [less than 6 mg/L]	52.9 ± 48.9	16.5 ± 9.7	0.099
ESR [up to 12 mm/h]	64.9 ± 32.5	49.2 ± 26.2	0.341
Montreal classification of CD [n (%)]			
Age at onset			
A2 (17–40 years)	2 (22.2%)	6 (100%)	0.007
A3 (>40 years)	7 (77.7%)	0 (0%)	0.007
Location			
L1 (Ileum)	4 (44.4%)	6 (100%)	0.044
L2 (Colon)	2 (22.2%)	0 (0%)	0.485
L3 (Ileocolon)	3 (33.3%)	0 (0%)	0.228
Behavior			
B2 (Stricturing)	3 (33.3%)	0 (0%)	0.228
B3 (Penetrating)	6 (66.6%)	6 (100%)	0.228
Perianal disease	1 (11.1%)	1 (16.6%)	1
Preoperative treatment [n (%)]			
Steroid	8 (88.8%)	3 (50%)	0.235
Azathioprine	5 (55.6%)	3 (50%)	1
Biological therapy	3 (33.3%)	5 (83.3%)	0.119
Operative findings [n (%)]			
Mass	3 (33.3%)	3 (50%)	0.622
Stricture	8 (88.8%)	5 (83.3%)	1
Obstruction	3 (33.3%)	3 (50%)	0.622
Perforation	0 (0%)	3 (50%)	0.044
Abscesses	0 (0%)	3 (50%)	0.044
Enteric fistula	3 (33.3%)	3 (50%)	0.622
Type of surgery [n (%)]			
Small intestinal RA and fistulectomy	2 (22.2%)	1 (16.6%)	1
Right Hemicolectomy	5 (55.6%)	4 (66.6%)	1
Sigmoid Colectomy	1 (11.1%)	0 (0%)	1
Sigmoid Colectomy and fistulectomy	1 (11.1%)	0 (0%)	1
Strictureplasty with fistulectomy	0 (0%)	1 (16.6%)	0.4
Features of surgery [n (%)]			
Intraabdominal sepsis	1 (11.1%)	2 (33.3%)	0.525
Temporary stoma	3 (33.3%)	2 (33.3%)	1
Abdominal surgery for other conditions	2 (22.2%)	2 (33.3%)	1
Surgery for CD recurrence	1 (11.1%)	1 (16.6%)	1
Postoperative treatment [n (%)]			
No	3 (33.3%)	0 (0%)	0.228
Azathioprine	4 (44.4%)	1 (16.6%)	0.580
Biological therapy	2 (22.2%)	5 (83.3%)	0.041

ALT, alanine aminotransferase; AST, aspartate aminotransferase; BUN, blood urea nitrogen; CD, crohn's disease; CRP, C-reactive protein; ESR, erythrocyte sedimentation rate; G1, Group 1 (postoperative recurrence of CD); G2, Group 2 (postoperative non-recurrence of CD); INR, international normalized ratio; RA, resection anastomosis; SD, standard deviation.

## 4 Discussion

IBD includes both UC and CD. These diseases are chronic inflammatory conditions which affect both morbidity and mortality of involved patients [15]. CD can affect any part of gastrointestinal tract with extraintestinal complications. CD has stricturing and penetrating behaviors which can lead to complications indicative for surgery [16]. In disease course of CD, surgery was needed in 40–70% of the patients [17,18].

Unfortunately, most patients with CD suffer from postoperative recurrence of the disease either clinical, endoscopic, serologic, or radiological recurrence. Re-operation was estimated to be about 50–60% of patients. Therefore, postoperative prevention is needed to reduce this rate of recurrence [19].

This retrospective study aimed to identify the characterizations of the CD in Egypt as there is a lack of data regarding the IBD patients in our country as well as most of the African countries, however with increased awareness and more development of diagnostic tools, more cases were diagnosed [4].

The literature provides controversial data for age as postoperative predictors for CD. Several studies showed that age was not a predictive factor for postoperative recurrence of CD [20,21]. The American Gastroenterological Association reported that patients <30 years had high probability rates of endoscopic and clinical postoperative recurrence as 80% and 50% respectively after 18 months [22]. In current study patients age >40 years (Montreal A3) showed statistically significant high postoperative recurrence.

Gender was not determined as risk factor for postoperative recurrence in current study. Studies in literature showed conflicting data, some of them determined male [23] or female [24] as a predictive factor for postoperative recurrence, while others showed no differences [20,25].

Like current results, different studies showed unreliability of serological markers for diagnosis of postoperative recurrence and were not predictive for clinical and endoscopic postoperative recurrence [26,27].

In current study, ileal location of CD was predictive factor for postoperative recurrence similar to several studies [28,29]. However, data in literature was found quite conflicting regarding this point as ileocolonic CD was found highly recurrent in Morar et al. retrospective study [30], while colonic CD was found highly recurrent in another study [31], and several other studies showed no relation between CD location and postoperative recurrence [20,32].

Regarding the penetrating behavior of CD, significant heterogeneity was found between different studies. Penetrating behavior of CD was associated with early postoperative recurrence, according to several studies [32,33]. But current study did not demonstrate significant differences of penetrating behavior of CD among recurrent and non-recurrent patients like others [9,34].

Also, stricturing behavior of CD was not significantly found in postoperative studied patients as reported also by Sachar et al. [33].

Many studies have focused on the effect of anastomotic configuration and fecal stream on postoperative recurrence rates. Fecal stasis, ileocolonic reflux, ischemia, and bacterial overgrowth may play a role in postoperative recurrence [35]. Rutgeerts et al. evaluated non recurrence of CD in patients with diverting ileostomy [36]. Wide lumen side-to-side anastomosis [37], and Kono-S end-to-end anastomosis [38] showed less correlation with postoperative recurrence. But other different studies showed no correlation between type of anastomosis and postoperative recurrence in agreement with current study [20,39].

The literature provides conflicting data regarding length of resected bowel and postoperative recurrence of CD. Several previous studies showed that the length of resected bowel was not consistently correlated with postoperative recurrence rate [40,41]. But Fazio et al., in 1996 changed this concept by showing that limited bowel resection margins from diseased bowel showed significant reduction of postoperative recurrence [42], and European Crohn and Colitis Organization has determined that bowel resection <50 cm could decrease postoperative recurrence [43]. In contrary, limited colectomy in current study showed no significant difference between postoperative recurrent and non-recurrent groups.

Many surgeons prefer conservative management of stricturing CD than stricturoplasty to avoid high rates of postoperative recurrences in these patients [44]. These high recurrence rates were equally recorded in conventional and non-conventional stricturoplasty techniques [45] and stricturoplasty with or without resection [46]. But currently different studies showed that strictureplasty could be done safely in properly selected patients with lower postoperative recurrence rates [47,48]. In current study, strictureplasty for a long bowel segment was conducted for one patient only without postoperative recurrence.

Recent European guidelines considered previous intestinal resection a risk factor for postoperative recurrence [43]. In current study only two patients had history of previous intestinal resection, one patient in each studied group. No statistically significant difference was found between them.

Current study demonstrated that postoperative treatment with anti-TNF agents could reduce the risk of postoperative recurrence in agreement with different previous studies [25,49]. In current study, patients used azathioprine postoperatively showed no significant difference in postoperative recurrence as reported by Yang et al. [50].

Smoking, history of surgery, perianal disease and penetrating behavior of CD are known risk factors for postoperative recurrence [22]. Current study did not show the same results regarding these risk factors which may be attributed to either the small number of patients in current study or racial difference which was not fulfilled researched in previous studies [25].

Limitation of current pilot study is that it was done retrospectively at a single academic center on a few numbers of patients prefer conservative treatment than surgical intervention which cannot cure their disease. So, larger future multicenter studies will be needed to assess current study findings.

## 5 Conclusion

Surgery can treat patients with complicated CD, however postoperative recurrence for those patients is still considered a problem. Predictors for recurrence can be helpful and some of them are preventable. Age at diagnosis, age at operation, ileal location of CD can significantly predict postoperative recurrence. Also, postoperative biological therapy can significantly decrease the incidence of postoperative recurrence.

## Ethical approval

The study was approved by Research Ethics Committee of Ain Shams University Faculty of Medicine corresponding to declaration of Helsinki principles (FMASU R 78/2021).

## Sources of funding

None.

## Registration of research studies

1 Name of the registry: Research Registry
2 Unique Identifying number or registration ID: researchregistry7022
3 Hyperlink to your specific registration (must be publicly accessible and will be checked): https://www.researchregistry.com/browse-the-registry#home/registrationdetails/61072ccecba81c001ef531f7/

## Guarantor

Y.E. and S. K.

## Consent

All studied patients approved their involvement in the study by written, informed consent.

## Provenance and peer review

Not commissioned, externally peer-reviewed.

## Author contribution

**Shimaa Kamel:** Study concept & design, data collection & analysis and writing the paper. **Mohamed Sakr, and Waleed Hamed:** revised manuscript for important intellectual content and approved the version to be published. **Mohamed Eltabbakh, Ahmed Sherief, and Heba Rashad:** data collection & analysis. **Yasser Elghamrini:** Study concept & design, data collection & analysis and performing surgical procedures. **Ahmed Elbaz:** Data analysis and writing the paper.

## Declaration of competing interest

Authors declare no conflict of interest.
